# Critical Dynamics Mediate Learning of New Distributed Memory Representations in Neuronal Networks

**DOI:** 10.3390/e21111043

**Published:** 2019-10-26

**Authors:** Quinton M. Skilling, Nicolette Ognjanovski, Sara J. Aton, Michal Zochowski

**Affiliations:** 1 Biophysics Program, University of Michigan, 930 N University Ave., Ann Arbor, MI 48109, USA; qmskill@umich.edu; 2 Department of Molecular, Cellular, and Developmental Biology, University of Michigan, 1105 N University Ave., Ann Arbor, MI 48109, USA; nnognjan@gmail.com (N.O.) saton@umich.edu (S.J.A.); 3 Department of Physics, University of Michigan, 450 Church St, Ann Arbor, MI 48109, USA

**Keywords:** memory and learning, memory consolidation, critical phenomena

## Abstract

We explore the possible role of network dynamics near a critical point in the storage of new information in silico and in vivo, and show that learning and memory may rely on neuronal network features mediated by the vicinity of criticality. Using a mean-field, attractor-based model, we show that new information can be consolidated into attractors through state-based learning in a dynamical regime associated with maximal susceptibility at the critical point. Then, we predict that the subsequent consolidation process results in a shift from critical to sub-critical dynamics to fully encapsulate the new information. We go on to corroborate these findings using analysis of rodent hippocampal CA1 activity during contextual fear memory (CFM) consolidation. We show that the dynamical state of the CA1 network is inherently poised near criticality, but the network also undergoes a shift towards sub-critical dynamics due to successful consolidation of the CFM. Based on these findings, we propose that dynamical features associated with criticality may be universally necessary for storing new memories.

## 1. Introduction

Phase transitions and critical phenomena are of central importance to statistical physics and there is growing evidence supporting its crucial role in living systems [1,2,3]. Here we investigate how near critical network dynamics may recruit neurons and facilitate formation of a new distributed memory in a situation where the incoming input must compete with the already stored (native) memories for neuronal resources. 

It is widely hypothesized that new information is encoded in brain circuits through activity dependent, long-term synaptic structural changes [4] that are a putative substrate for memory formation [5,6]. While features of memory traces can be localized to specific cell populations (e.g., location information encoded in place cell activity), in general, tracing so-called “engrams” to neural circuits has been an elusive task [5]. Attempts at disrupting well-established memories through brain lesions [7] or, more recently, through optogenetic silencing [8] have shown that they are robust to alterations in communication between individual neurons or brain areas. A parsimonious and longstanding explanation of these phenomena is that a process termed “systems consolidation” leads to diffuse, widespread memory encoding and storage. However, despite more than a century of study, it is not well understood how engrams are initially formed and subsequently stored across vast distances (in terms of numbers of synaptic connections between neurons) in the brain.

A major problem to understanding the mechanisms for systems consolidation is that very little is known about how the formation of new memories (i.e., learning) impacts neural network dynamics. The general, long-accepted assumption is that either strengthening of existing synaptic connections, or the de novo creation of additional synapses (i.e., formation of a discrete structural heterogeneity) leads to the formation of a dynamical attractor [9,10]. If this is the case, then the dynamical state of the network must support long-range correlations across the network, as the number of neurons actively involved in encoding a specific memory trace is thought to constitute only a small fraction of the total neuronal population [11]. Moreover, individual synapses in regions such as the hippocampus have a surprisingly brief lifetime (approximately 1–2 weeks on average [12]), necessitating rapid dissemination and consolidation of information. These requirements raise two questions: (1) how do permanent and widely-distributed neural engrams form from initial, transient changes to a discrete subset of the network’s synapses during learning, and (2) what mediates transformation of local representations of disparate features to global memory representation? New experimental [13] and computational work shows that theta band oscillatory patterning and/or dynamics associated with sharp wave ripples can mechanistically coordinate neuronal activity recruiting them into the representation [14]. 

In this work, we show computationally that in addition to large scale temporal pattering of neuronal activity, near-critical dynamics in the brain could be an important factor in facilitating memory consolidation. Specifically, we show that storage of new information that is weakly and/or sparsely impinged on the network is mediated through plastic, state-dependent changes in network connectivity and can be successfully consolidated (which is associated with attractor formation) near criticality—a point associated with second order phase transitions [15]. This storage is followed by a subsequent shift from critical to sub-critical dynamics. 

The idea that the brain operates at or near dynamical critically is not new (see References [1,16,17] for comprehensive reviews) and it was experimentally observed in in vivo and in vitro preparations [18,19,20,21,22,23,24,25,26,27]. A large body of work also investigated the potential functional benefit of operating in a near-critical regime [28,29,30,31,32]. Here, we specifically identify very basic, underlying importance for the brain to reside near criticality and demonstrate that near-critical dynamics may be essential for a system-wide consolidation of new memories in a situation when the sensory input is weak and/or sparse in comparison with signals generated by memories native to the network (i.e., those previously stored). 

To substantiate these hypotheses, we analyze in vivo recordings associated with contextual fear memory (CFM) consolidation. Contextual fear conditioning (CFC) is an optimal experimental paradigm in this regard as it allows for rapid formation and consolidation of memory (i.e., after single-trial learning) [13,33]. In this particular case, the CFM consolidation is associated with normal sleep, which has been shown to play a vital role in various types of memory consolidation [13,33,34,35]. Here, we first characterize hippocampal dynamics in mice subjected to CFC and show that: (1) the hippocampus operates in a near critical regime pre- and post-CFC training, and (2) successful, behaviorally-verified consolidation of fear memory leads to an underlying shift in hippocampal dynamics towards a subcritical state, similar to what we predict in our model simulations.

Together, these results indicate that novel learning may occur preferentially near a critical regime and leads to universal widespread stabilization of network activity patterns, which in turn drives the formation of widely-distributed engrams (i.e., systems memory consolidation). 

## 2. Consolidation of New Memory Near Criticality in Attractor Neural Networks

We modeled a neuronal network with easily controllable dynamics using a mean-field, Hopfield-like formalism [10]. In this context, instantaneous neuronal states are modeled as binary variables, $S_{i}=\pm 1$, corresponding to a firing (+1) and a quiescent (−1) neuronal state, respectively. Instantaneous states are updated based on a neuron’s input

(1) $$h_{i}=\frac{1}{k}\sum_{j=1}^{k}J_{ij}S_{j}$$

which serves to align that neuron’s state with that input, so that $sgn\left(S_{i}\right)=sgn\left(h_{i}\right)$ with probability

(2) $$P\left(h_{i},\beta \;\right)=\;\frac{1}{1+exp\left(-2\beta \left|h_{i}\right|\right)}$$

where k is the incoming degree of each neuron, sgn(x) is the sign function, and J is the connectivity matrix, discussed in detail below. The term $\beta =\;T^{-1}$ is a control parameter which directly controls the dynamical state of the system: when $\beta \ll 1,\;P\left(h_{i},\beta \right)\to \frac{1}{2}$ and, conversely, $\beta \gg 1,\;P\left(h_{i},\beta \right)\to 0$, with the critical point typically located near T= 1. These dynamics describe properties of the standard Hopfield model in the absence of an external field [36,37].

The network we use here consists of N=10000 neurons, arranged in a directional, small-world network (10% chance of rewiring a local connection) [38] with ~2% incoming connectivity, but with no self-connections allowed. Initially the network is seeded with p native memories (hereafter collectively referred to as the native state and designated by the superscript n) defined by a random configuration of states $\left\{\xi_{i}^{n}=\pm 1\;|\;i\;\in \left[1,\;\;N\right]\;\right\}$ for each memory, and with the weighted connectivity matrix indices defined as

(3) $$J_{ij}=\;\frac{1}{p}\sum_{\mu =1}^{p}\xi_{i,\mu }^{n}\xi_{j,\mu }^{n},$$

for all incoming connections (hence, $J_{ii}=0$).

With p memories already embedded in the network through Equation (3), we want to investigate how the network responds to, and possibly consolidates, a new representation with randomly configured states similar to the native memories,$\;\left\{\xi_{i}^{e}=\pm 1\;|\;i\;\in \left[1,\;\;N\right]\;\right\}$ (the superscript e hereafter representing a configuration of states associated with the new memory). However, throughout evolution of the network the new representation influences only a small subset, $N^{\prime }$, of network neurons; here, the neurons belonging to $N^{\prime }$ are randomly selected from the full network. The instantaneous states of these neurons do not change throughout the simulation and are set to $S_{i}\left(t\right)=\xi_{i}^{e},\;\forall \;t,i\in N^{\prime }$. In addition, a connection emanating from these input neurons is modified to:

(4) $$J_{ij}=\;\frac{\;w^{e}}{p}\xi_{i}^{e}\xi_{j}^{e}\;\forall \;j\in N’,$$

where the $w^{e}$ term is the additional weight of the connections corresponding to the new state relative to the native connectivity (Equation (3)). We formulated the input in this way to mimic real biological processes of learning and memory. The subset of the input neurons and their corresponding connectivity is to roughly represent the memory backbone formed rapidly during the presentation of the new input (associated with the new representation). At the same time the freezing of the dynamics of these neurons is to correspond to input constancy during the experience, or, reactivation of these neurons during sleep that was observed experimentally [39,40].

Although the majority of neurons in the network encode for the native memories, those receiving input from neurons representing the new state will align with it if the new state is fractionally stronger than the native state at any time. The competition between the native and new states are encapsulated in the total input a neuron receives,

(5) $$h_{i}\left(t\right)=\sum_{j\notin N^{\prime }}J_{ij}S_{j}\left(t\right)+w^{ext}\sum_{l\in N^{\prime }}J_{il}\xi_{l}^{e}\equiv h_{i}^{n}\left(t\right)+h_{i}^{ext},$$

where $h_{i}^{n}\left(t\right)$ is given by Equation (1) and $h_{i}^{ext}$ represents input from the constant external field, here facilitated by fixed neuron states.

We assessed the presence of attractors in the network by measuring the overlap of the final state of the network with one of the native configurations ($m_{\mu }^{n}=\left|\frac{1}{\left|N-N^{\prime }\right|}\sum_{i\in N-N^{\prime }}\xi_{i,\mu }^{n}S_{i}\right|$) and/or the new configuration ($m^{e}=\left|\frac{1}{\left|N-N^{\prime }\right|}\sum_{i\in N-N^{’}}\xi_{i}^{e}S_{i}\right|$), where the averages are over all non-fixed neurons in the network (i.e., the relative compliment of N and N’). 

**Figure 1:**
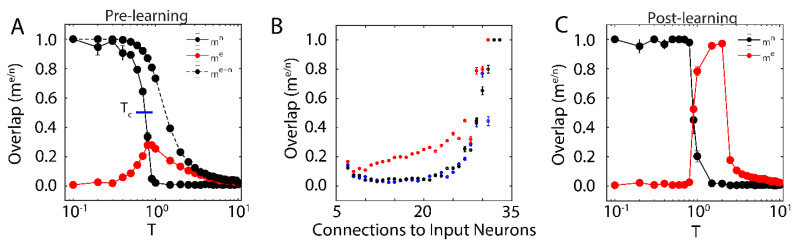
New memory consolidation occurs only near criticality. (**A**) Overlap of the system with the native configuration without external input (solid black line) and with external input (dashed black line), as well as overlap with the new configuration (red) represented by external input, as a function of temperature before learning. Note that maximal susceptibility of the new configuration only occurs near the initial critical temperature of the system, where overlap with the native configuration declines. Here, we define the critical temperature to be the temperature where the order parameter (Overlap) reaches its half-maximal value, as indicated by the blue line. (**B**) Overlap of the new configuration after learning for neurons grouped based on their number of connections to the input. Colors represent pre-learning sub- (blue) super- (black) and critical (red) temperatures. (**C**) Overlap of the system with the native (black) and new (red) configurations as a function of system temperature after learning. Few changes in overlap occur before the initial critical temperature, after which (near criticality) the system aligns to the new configuration. Note also that the new configuration overlap occurs for larger values of temperature, indicating consolidation and a shift in critical temperature due to learning. All error bars in (**A**–**C**) represent the standard error of the mean.

First, we examined the overlap of the network with the new configuration (red solid line in Figure 1A; with $\left|\;N^{\prime }\right|=700$) and the native ones (black solid line in Figure 1A) when only one native memory is stored in the system (p=1) as a function of temperature T. The universal dynamical properties of the system at criticality maximizes the susceptibility to the external input at the critical point. In this work, we define $T_{c}$ as the point where the order parameter (i.e., $m_{\mu }^{n}$) reaches a half-maximal value (when quantified, it is found via linear interpolation between point pairs). This point coincides with the half maximal value achieved by stability, another order parameter of the system (see below). Here, the critical point has been well-characterized as a second order phase transition that separates the phase of high-stability dynamics ($T<T_{c}$; characterized by convergence to stored attractors) from disordered dynamics ($T>T_{c}$) [37]. The result is a well-defined regime, where the network overlap with the new configuration, impinged on the system through external input, is higher than that of native memories. When the system is sub-critical ($T<T_{c}$), the overlap with one of the native memories dominates the system. In contrast, the super-critical network ($T>T_{c}$) is in a disordered state where neither the native configuration nor the new configuration dominates dynamics. At ($T\sim T_{c}$) the attractor associated with native memory becomes unstable, and at the same time magnetic susceptibility peaks making the overlap of the network with the new representation significantly higher. However, if the states of the input neurons are set to the values of the natively stored configuration, i.e., $\xi_{i}^{e}=\;\xi_{i}^{n},\;i\in N^{\prime }$ (during a memory recall event, for example), the stability of the native memory is extended over the critical range (Figure 1A dashed black line), shifting the phase transition towards higher temperatures. Thus, depending on the input configuration, at ($T\sim T_{c}$) both, native memory can be stabilized or new memory representation can be fractionally successfully impinged on the system. This theoretically provides the network with agility to store a new memory or to retrieve a known one. Such shift away from criticality in presence of structured input was also observed in self organizing recurrent networks (SORNs [41]), and may explain slightly subcritical brain states observed in vivo [27].

We next wanted to investigate how proximity to the external input (through numbers of connections) effects the corresponding overlap, $m^{e}$ for different temperature ranges. We measure fractional overlap of the final network state with the new configuration as a function of the number of connections that neurons receive from the external input neurons (Figure 1B); those neurons receiving higher native input should align with the native configuration, whereas neurons with higher non-native input should be driven to adopt the new configuration (under the right dynamical state, given by the control parameter β). We observe, as predicted, that the mean overlap of neuronal states with the new configurations is significant and highest (Figure 1B, red curve) for neurons receiving the external input at criticality, as compared to sub-critical (black points), and super-critical (blue points) regimes. Thus, at criticality, as opposed to sub-critical and super-critical regimes, even sparse and/or weak input can lead to global changes in the network, providing a plausible mechanistic explanation for the distributed nature of memory traces.

We next investigated whether application of a type of activity-dependent synaptic plasticity rule observed experimentally [42] can lead to consolidation of the new configuration. Here by consolidation we mean whether (a) the overlap between the new configuration and stability of the network in the presence of input can be increased, and (b) whether the stable (in absence of the external input) attractor representing the new configuration can be successfully formed. 

We implement these synaptic changes in the model by introducing state-based changes in connectivity strengths,

(6) $$\Delta J_{ij}\left(t\right)=\varepsilon S_{i}\left(t\right)S_{j}\left(t\right)\;.$$

During the learning phase, both the neural states and the connections were updated (with ε=0.1), with the exception of neurons pertaining to the external input (i.e., those neurons remain fixed and so receive no relevant input). 

We investigated the range of the control parameter, T, for which the network is able to successfully store the new configuration (i.e., the emergence of a new attractor with a large value of $m^{e}$). We found that the system successfully consolidated the new configuration starting near $T_{C}$, indicated by an increase in $m^{e}$ post-learning (Figure 1C). This shows that new memory consolidation occurs only near criticality, when susceptibility to external input drives the increased overlap with new configuration (Figure 1C). In addition, we observed that consolidation shifts $T_{C}$ to higher values of T, causing an initially critical regime to become sub-critical. These changes (due to the unbounded learning rule) lead to an increase of the overall magnitude of synaptic coupling, resulting in a stronger external field and ultimately leading to a peak in $m^{e}$ at $T>\;T_{C}$ after learning.

**Figure 2:**
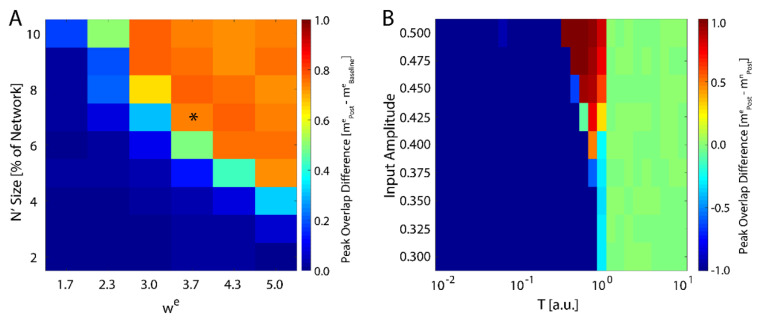
Robustness of new memory consolidation as a function of input strength. (**A**) Peak change in overlap of the new state between pre- and post-learning as a function of input size (percentage of fixed nodes in the network) and strength ($w^{e}$). The asterisk represents the parameters used to generate the data showed in Figure 1. (**B**) Change in overlap (color) between the new and native configurations post-learning as a function of temperature for increasing values of external field strength applied during learning. Blue colors represent cases where the native configuration is still stable after learning, red colors are where the new configuration is stable, and green is where neither configuration is stable. Note that for sufficiently high field strength, we see a slight increase in the maximal critical temperature.

We next investigated how the consolidation depends on the number of input neurons, i.e., the size of $N^{\prime }$, and the magnitude of the weight of the connections stemming from the input (Equation (4)). We varied both of these parameters and monitored maximal change in magnitude of new-state overlap from pre-learning (as exemplified on Figure 1A) to post-learning (Figure 1C). These results are presented in Figure 2A and one can observe that the number of input neurons can be as small as 4% of the total network size to observe meaning full change in the overlap over the rest of the network. Conversely the $w^{ext}$ can be as low 2.3 to observe increase of the overlap. Hence, even weak and sparse input can have noticeable impact on network dynamics, but only near criticality. The asterisk represents the parameter configuration used to generate results presented on Figure 1. 

Up to this point, we have examined network response to external input represented by fixed neuronal states in the network. Alternatively, instead of the new memory being represented by specific neurons, we can represent the new memory as persistent input to all neurons in the network by defining an extra term for the observed input,

(7) $$\begin{array}{c} h_{i}\left(t\right)=h_{i}^{n}\left(t\right)+h_{i}^{ext}\\ h_{i}^{ext}=\;\frac{w^{ext}}{p}\xi_{i}^{e} \end{array}$$

with $h_{i}^{n}\left(t\right)$ again being represented by Equation (1). We ran the simulation in the presence of a fixed external field applied to all neurons with learning (a pre-learning phase followed by a learning phase), followed by an additional phase with $h_{i}^{ext}=0$ and then subsequently calculated the difference in the final overlap between the new and the native memory. We found that the system only consolidates the new configuration given sufficiently high external field strength, and only near the critical temperature (Figure 2B). Hence, the system is able to adapt to the new configuration, regardless of its source, only near the critical regime, in support of previous studies [43]. Importantly, higher field magnitude increases the range of temperatures for which the new memory is consolidated (top of Figure 2B).

**Figure 3:**
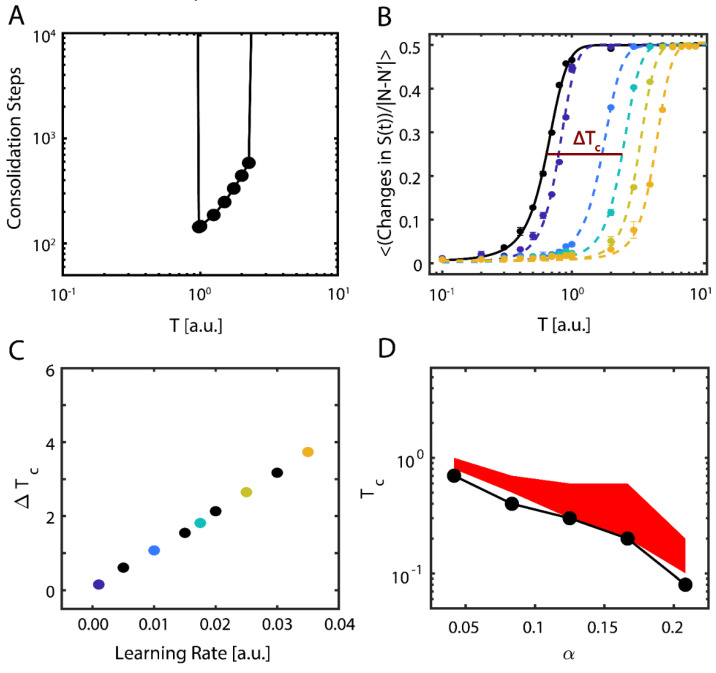
Dynamical properties of consolidating new information. (**A**) Time (steps) required for the system to consolidate the new configuration, as a function of temperature. Values not shown (on the left and right sides) indicate timescales longer than the simulation runtime, *i.e.* that it takes a prohibitively long time to consolidate a new memory. (**B**) Data and fit sigmoidal functions for mean number of changes in the neurons’ state *S_i_* per iteration as a function of temperature, pre- (solid black line) and post-learning (dashed lines); the learning rate ε increases left-to-right and from darker to lighter colors of the dashed lines. Error bars represent the standard error of the mean. The horizontal line labeled Tc represents the half-maximal point of the where we calculate the critical temperature via linear interpolation. (**C**) Change in observed critical temperature $T_{c}$, calculated using the sigmoidal half-maximum values (**B**) as a function of the learning rate ε. Colors correspond to the curves shown in (**B**). (**D**) Critical temperature $T_{c}$ as a function of the memories per degree distribution α before (black points) and after (red shaded region) learning for a new-state connectivity strength of $w^{e}=3.0$. Note that the minimal value of the critical temperature for the new configuration post-learning closely matches the critical temperature pre-learning, but that the effect of learning is a broadening of the stable regime.

These results show evidence of the possible importance of near-critical dynamics in storing new memories. To be more robust with our findings, we further examined the properties of new memory consolidation. We first calculated the amount of time (i.e., the number of iterations) needed for the network to align with the new configuration, so that $m^{e}\geq \;\frac{1}{2}$ (Figure 3A). Near the initial (i.e., pre-learning) value of $T_{C}$, only a fraction of the learning time was required to consolidate the new configuration, and with increasing T, the consolidation time increased exponentially before abruptly increasing to an interval greater than the simulation time. In contrast, sub-critical and super-critical states were marked by prohibitively long consolidation periods (left- and right-hand sides of Figure 3A, respectively). 

Next, we examined how changes to the learning rate (ε in Equation (6)) affects both the consolidation of the external input representing the new configuration and the dynamical properties of the system. To assess the transition point (i.e., $T_{C}$), we measured the network’s configuration stability, f(T), as a function of temperature for different values of ε. Stability here is defined as the mean number of changes from active (+1) to quiescent (−1) states occurring in the network for fixed simulation length; the expected number of these activity changes is 0 in the sub-critical regime and ~N/2 in the super-critical regime, due to Equation (2). We subsequently fit sigmoidal functions to the transition numbers as a function of temperature, taking the form $f\left(T\right)=\frac{0.5}{1+exp\left(-\frac{T-T_{c,i}}{\mu }\right)}$, where the slope μ represents the change in stability due to changing regime and we designate $T_{c,i}$, the temperature where the transitions reach their half-maximum value, as a proxy for critical temperature (Figure 3B); as previously mentioned, f(T) is thus an order parameter of the system. We next calculated the change in the critical temperature due to learning, $\Delta T_{c,i}=T_{c,\;i}^{\;}\left(t_{0}\right)-T_{c}^{\;}\left(t_{final}\right)$, and found that consolidation of new information shifts the stability, and therefore the critical regime, of the system approximately linearly with the learning rate (Figure 3C).

Finally, we investigated the behavior of the system when it is loaded with multiple native configurations, and when the location of the critical point is a function of both memory loading α and temperature T [36]. We thus pre-loaded additional native configurations into the network. It is known that memory recall fails for T = 0 at $\alpha_{max}=\frac{p_{max}}{N}\sim 0.14$ (with $p_{max}$ being the maximal number of configurations stored and N number of neurons in the network) for a fully connected network [37], but this value changes for a sparsely connected system and is proportional to nodal degree k, $\alpha_{max}=\frac{p_{max}}{\langle k\rangle }$. 

We found that regardless of the number of memories pre-loaded into the system (below the loading limit), successful consolidation of new configuration always occurs near $T_{C}$ (Figure 3D). Here, the black curve represents the location of the pre-learning critical point, estimated as the first point where rapid decline of stability for the native configuration occurs (i.e.,$\;m^{n}<0.45$), whereas the red area is the parametric space where the new memory is consolidated ($m^{e}\geq 0.45$).

Taken together, the model simulations outline how the process of learning is affected by dynamics near criticality. Here, the system is highly susceptible to network input and subsequently consolidates new configurations through state-based plastic changes in network connectivity strengths. If, on the other hand, the input corresponds to one of the native memories their stability is extended over the critical temperature range. Thus, the critical state on one hand provides metastability to native configurations allowing their retrieval in presence of correct external input, but also provides dynamical substrate for storage and consolidation of the new configurations.

Further, during learning, the synaptic plasticity shifts the critical point, extending the sub-critical regime post-learning. To test whether these are general principals of learning in neuronal networks in vivo, we next analyzed spike data recorded from neurons in mouse hippocampal area CA1 during consolidation of a fear memory. 

## 3. Consolidation of a Fear Memory Results in Subcritical Neural Dynamics in the Mouse Hippocampus 

We analyzed spiking data recorded from hippocampal area CA1 of mice subjected to contextual fear conditioning (CFC) in order to investigate the effect of learning on network dynamics. Specifically, mice are placed in the novel environment that they are allowed to explore briefly. They are subsequently exposed to electric shock while in the novel environment (induction of CFC) or not (sham) through the wire mesh placed in the floor. The mice exposed to the shock exhibit a freezing behavior (i.e., they stop moving) in the novel environment on subsequent presentation while the sham mice do not. Using CFC, long-lasting fear memories (CFMs) can be successfully consolidated in mice in the hours following a single training trial, consisting of placement in a novel environmental context paired with a foot shock. This single-trial learning, unlike more elaborate training procedures (e.g., object recall or track learning), provides clear boundaries between baseline and post-conditioning and allows for direct comparisons of network dynamics. Further, memory consolidation in general [35] and fear memory consolidation in particular [13,33,34] is known to rely on sleep, a vigilance state characterized by internally driven dynamics and thus allowing the possibility for truly self-organized neural behavior [44]. 

**Figure 4:**
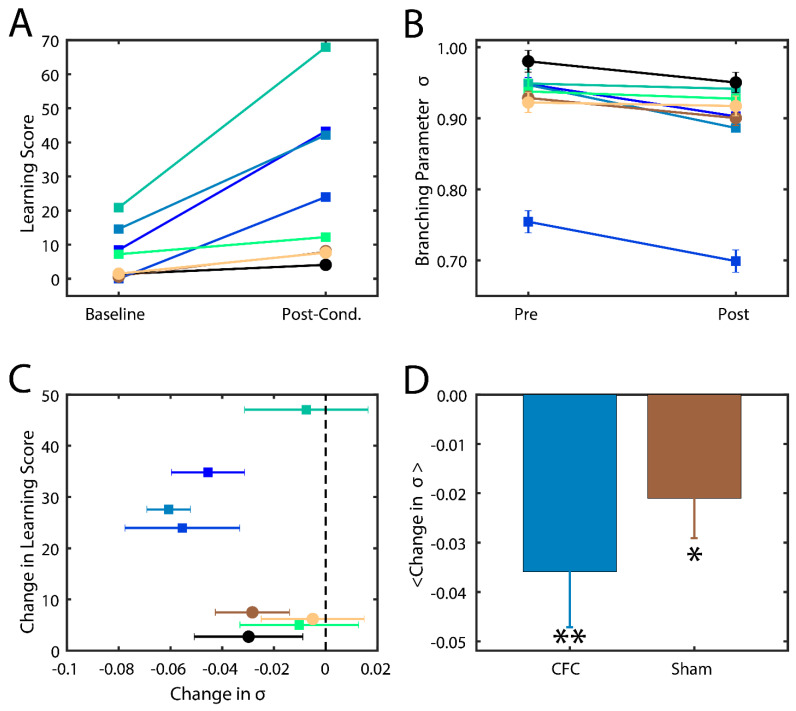
Branching parameter and its changes as a function of quality of memory consolidation during SWS sleep. (**A**) Percentage of freezing behavior observed in mice before (baseline) and after learning (post-cond.) for sham (circles) and CFC (squares) groups. Different colors represent different mice. (**B**) Branching parameters σ during SWS before (baseline) and after learning (post-cond.). Colors and shapes are conserved as in (**A**). Error bars represent the standard error of the mean, calculated for each mouse over all intervals. (**C**) Change in freezing behavior vs change in branching parameter across the learning interval. Error bars represent the propagation of standard errors between Pre and Post in (**B**). (**D**) Mean change in branching parameter within each group. Error bars represent the standard error of the mean. * p < 0.10 confidence interval that the reduction was significant; ** p < 0.02 confidence interval that the reduction was significant, using the one-way T test.

Successfully consolidated CFMs manifest as visual changes in behavior, where mice cower in place (i.e., freezing behavior) instead of adopting their normally inquisitive or explorative nature [13,33]. The level of success of memory consolidation is quantified by a percent change in this behavior as compared to baseline, which we hereafter refer to as the learning score. In this study, we thus compare the behavior and analysis of hippocampal recordings across two groups of mice: (1) contextual fear conditioned mice (CFC) that are given a fear stimulus in a novel environment and have ad lib sleeping patterns in the 24 h following the stimulus; and (2) sham mice that are introduced to the novel environment but do not receive a foot shock and are not sleep deprived. More information about the experimental procedure can be found in the Methods section. We indeed found that CFC mice had higher learning scores post-stimulus compared to their Sham counterparts (Figure 4A).

In order to substantiate our model hypothesis that (a) near-critical dynamics may be important for memory consolidation and (b) that consolidation actually stabilizes the system, CA1 neurons’ spiking data was analyzed for proximity to a critical state by calculating the branching parameter [18]. While other metrics have been used to determine dynamical states, namely, power-law-distributed avalanches [18,44], the benefit of the method described here is that it better controls for spurious correlations between the data and can account for slowly varying dynamical changes [45]. A previous study by the Priesemann group addressed this issue by showing a more accurate branching parameter can be determined by taking into account the relationship between the variance and covariance of the branching parameter and by eliminating data sets that showcase non-stationarities [45]. In this study, we used the python package associated with their study to calculate the optimal branching parameter σ (Python Package Index—mrestimator v 0.1.4; https://pypi.org/project/mrestimator). For each Slow Wave sleep (SWS) interval, we binned hippocampal spiking data into sub-intervals of 16 ms, calculated the avalanche size (i.e., the number of spikes) in each interval, then used the provided software to calculate the branching parameter. Data sets that failed tests of non-stationarity (e.g., due to fast fluctuation between up and down states or from external drive; see [45]) were removed, and the average branching parameter was calculated. 

We calculated σ from CA1 spike data recorded in the two groups (CFC and Sham) from every bout of SWS during 24 h time interval post CFC. We analyzed only SWS as during wake the mice are constantly swamped with new input making assessment of intrinsic hippocampal dynamical state impossible. At the same time rapid eye movement (REM) sleep bouts in mice are few and short in duration making branching parameter estimate unstable (i.e., it failed many criteria set forth in [45]). We found that mice in both groups had branching parameters near σ = 1 (Figure 4B), indicating that the mouse brain naturally has near-critical dynamics. After the learning interval, we observed a noticeable decrease in σ in most CFC compared to Sham mice (Figure 4C). Indeed, we found that an increase in learning score generally exhibited a decrease in their branching parameter (Figure 4C) away from a critical state and that CFC mice exhibited a more significant reduction due to learning (*p* < 0.02) compared to Sham animals (*p* < 0.10). These data indicate that (a) typical in vivo dynamics lie near criticality and that (b) consolidation of memory in vivo causes a deviation from critical to sub-critical behavior, as predicted by modeling. The smaller drop in branching parameter in sham group may also be associated with (smaller) degree of consolidation of the new environment even without the electric shock. 

## 4. Discussion

The question we address in this paper is how relatively sparse input can dynamically compete with already stored representations, to be stored and later consolidated into a distributed memory (engram). Through computational modeling work and analysis of in vivo hippocampal recordings, we show that criticality may play a pivotal role in mediating stabilization and subsequent storage of the new memory as a distributed representation. Namely, we show in a reduced attractor network, that only when the system is near a critical point can the new representation globally impinge its activity pattern on the network, making it fractionally dominant as compared to the native representation (Figure 1). This is primarily due to the fact that at criticality, when the system has the highest susceptibility to the external input, this input biases the state of the network towards the new representation, and the emergence of long-distance correlations allows it to spread throughout the system. Subsequently, state dependent synaptic plasticity allows for long-term storage (consolidation) of this new representation, even as it competes with a broad range of native configurations (Figure 3D). Thus here, similarly to results shown in self-organizing recurrent models [41], presentation of organized input results in a shift in the parametric location of criticality (Figure 1B), due to increased stability of native representation or storage of the new representation (Figure 3B). 

We thus hypothesize that criticality on one hand provides metastability to already stored configurations, so that if a native memory is presented through input, the memory is retrieved via the stabilized attractor, while on the other hand criticality provides a dynamical substrate for storage and consolidation of the new representations.

We find that successful new memory consolidation possibly changes the underlying dynamical state from being near-critical to being slightly sub-critical (Figure 3). Previous studies have reported a similar, slightly sub-critical dynamical state of the brain [27] which here seems to be the result of system consolidation to new information. Indeed, we see a similar deviation from critical to sub-critical dynamics in hippocampal recordings of mice successfully consolidating fear memories in vivo (Figure 4). This phenomenon can be explained as follows: before learning, susceptibility to external input is maximal near a critical point but, as learning commences, the system adapts by strengthening the connectivity to consolidate this new information extending the region of dynamical stability. 

Our results indicate that the brain operates near criticality, possibly slightly sub-critical, and that plasticity plays an active role in reducing the dynamical state away from criticality during learning and consolidation. This is an agreement with previous work that suggests slightly sub-critical dynamics still impart increased tunability, response to external input, and long-range spatial and temporal correlations [46]. The extension of the Hopfield model we present here suggest that the critical point is indeed shifted (Figure 3B), rather than the phase transition region is widened, what would be indicative of emergence of Griffith phase [47]. However, some of our unpublished results obtained in models of self-organized criticality, which are similar to integrate and fire models, suggest that the critical point may indeed expand suggesting emergence of Griffith phase (data not shown).

This raises an interesting question: how does the brain finally reset to a near-critical state after learning, so that another (new) memory can be consolidated? Our work here does not address this issue, but previous work by others has shown that neurons and networks in the brain have built-in homeostatic mechanisms which serves to recalibrate synaptic efficacies (see References [48,49,50,51,52]), a process that was proposed to happen also during development [53]. Thus, it could be that homeostatic plasticity together with reduced external input during sleep is sufficient to drive the system towards criticality, as shown by Zierenberg, J. et al. [54]. Indeed, our in vivo analysis indicates that the role of sleep is not purely homeostatic [52], but instead involves active learning processes, in line with previous reports [40,55]. As an additional consideration: both the model system and mice subjected to fear stimuli involve relatively strong inputs to be learned. In processes that occur over longer time periods, the dynamical shift may be weak compared to homeostatic dynamical rescaling, making it hard to detect on such short time scales as we show here. Future work should thus be done to investigate the interplay between homeostatic-based and learning-based changes in system dynamics near criticality.

### Experimental Methods

Male C57BL6/J mice (Jackson, aged 2–5 months) were implanted with driveable headstages containing two bundles of 7 stereotrodes each (spaced 1 mm apart) for single-unit and local field potential (LFP) recordings, and silver-plated wires for nuchal electromyographic (EMG) recording. LFP and EMG signals were used to assign behavioral states (wake, NREM, and REM sleep) in 5 s epochs throughout the recording period. Mice were individually housed (in standard caging with beneficial environmental enrichment including nesting material, manipulanda, and treats) during post-operative recovery and subsequent behavioral experiments. Lights were maintained on a 12 h:12 h light–dark cycle, and food and water were available ad lib, throughout all procedures. All housing and experimental procedures were approved by the University Committee on Use and Care of Animals at the University of Michigan. 

Following a 1-week recovery period, mice were habituated to daily handling (5–10 min/day) for 3 days. During this habituation period, stereotrodes were gradually lowered into CA1 until stable neuronal recordings (with characteristic spike waveforms continuously present on individual recording channels for more than 24 h) were obtained. Electrode positions remained fixed throughout subsequent experimental procedures. All mice underwent a 24 h baseline recording starting at lights on (9 AM). 

At lights on the following day, mice underwent single-trial contextual fear conditioning (CFC, *n* = 5) or sham conditioning (Sham, *n* = 3) [33]. Mice were placed into a standard conditioning chamber (Med Associates) with patterned Plexiglass walls and a metal grid floor. All mice were allowed to freely explore the novel chamber over the 3-min training session; CFC mice (but not Sham mice) received a 2 s footshock (0.75 mA) after the first 2.5 min. At the end of 3 min in the conditioning chamber, mice were returned to their home cage for an additional 24 h recording period. After 24 h following training, at lights on, mice were returned to the conditioning chamber for a 5 min assessment of contextual fear memory. This was calculated as the change in context-specific freezing (cessation of all movement save respiration), referred to as the learning score, between testing and training trials (i.e., percentage of time spent freezing at test—percentage of time spent freezing, pre-shock, at baseline).

Electrophysiological signals recorded from the hippocampus before and after CFC or Sham conditioning were digitized and differentially filtered as spike and LFP data as described previously [33] using Omniplex hardware and software; single-unit spike data was discriminated using Offline Sorter software (Plexon). The firing of individual neurons was tracked throughout each experiment on the basis of spike waveform, relative spike amplitude on the two stereotrode recording channels, positioning of spike wave-form clusters in three-dimensional principal component space, and neuronal subclass (e.g., FS interneurons vs. principal neurons). Only those neurons that were reliably discriminated and continuously recorded across both the 24 h baseline and 24 h post-conditioning recording periods were included in subsequent analyses.
